# Long Non-Coding RNA MAGI2-AS3 is a New Player with a Tumor Suppressive Role in High Grade Serous Ovarian Carcinoma

**DOI:** 10.3390/cancers11122008

**Published:** 2019-12-12

**Authors:** Priyanka Gokulnath, Tiziana de Cristofaro, Ichcha Manipur, Tina Di Palma, Amata Amy Soriano, Mario Rosario Guarracino, Mariastella Zannini

**Affiliations:** 1 IEOS - Institute of Experimental Endocrinology and Oncology ‘G. Salvatore’, National Research Council, via S. Pansini 5, 80131 Napoli, Italy; priyanka.gokulnath@gmail.com (P.G.);; 2 Dpt. Environmental, Biological and Pharmaceutical Sciences and Technologies, University of Campania “Luigi Vanvitelli”, 81100 Caserta, Italy; 3 High Performance Computing and Networking Institute, National Research Council, via P. Castellino 111, 80131 Napoli, Italy; 4 Present affiliation: IRCCS Casa Sollievo della Sofferenza, Cancer Stem Cells Unit, ISReMIT, 71013 San Giovanni Rotondo FG, Italy

**Keywords:** lncRNA MAGI2-AS3, High Grade Serous Ovarian Cancer, Epithelial Ovarian Cancer, Fallopian Tube, competing endogenous RNA, microRNA, non-coding RNA, tumor suppressor, RNA-sequencing analysis

## Abstract

High-Grade Serous Ovarian Carcinoma (HGSC) is the most incidental and lethal subtype of epithelial ovarian cancer (EOC) with a high mortality rate of nearly 65%. Recent findings aimed at understanding the pathogenesis of HGSC have attributed its principal source as the Fallopian Tube (FT). To further comprehend the exact mechanism of carcinogenesis, which is still less known, we performed a transcriptome analysis comparing FT and HGSC. Our study aims at exploring new players involved in the development of HGSC from FT, along with their signaling network, and we chose to focus on non-coding RNAs. Non-coding RNAs (ncRNAs) are increasingly observed to be the major regulators of several cellular processes and could have key functions as biological markers, as well as even a therapeutic approach. The most physiologically relevant and significantly dysregulated non-coding RNAs were identified bioinformatically. After analyzing the trend in HGSC and other cancers, MAGI2-AS3 was observed to be an important player in EOC. We assessed its tumor-suppressive role in EOC by means of various assays. Further, we mapped its signaling pathway using its role as a miRNA sponge to predict the miRNAs binding to MAGI2AS3 and showed it experimentally. We conclude that MAGI2-AS3 acts as a tumor suppressor in EOC, specifically in HGSC by sponging miR-15-5p, miR-374a-5p and miR-374b-5p, and altering downstream signaling of certain mRNAs through a ceRNA network.

## 1. Introduction

Epithelial Ovarian Cancer (EOC), accounting for nearly all of the ovarian cancers (OC) [1], is the most lethal gynecological malignancy [2]. It is the eighth most incidental and the most common cause of death in women [3], with about half the patients diagnosed with the disease succumbing to it within the next 5 years [4,5]. The study conducted by GLOBOCAN predicts the incidence and mortality will go up by 55% and 67%, respectively [3]. High-Grade Serous Ovarian Cancer (HGSC), one of EOC subtypes, accounts for around 70% of incidence and 65% mortality of all ovarian cancers [6,7]. This is attributed to very late diagnosis, almost always after the disease metastasis, often showing an abdominal spread [8]. The patients even after surgery and chemotherapy mostly succumb to the disease within 5 years mainly due to acquired therapeutic resistance [5]. The late diagnosis is because of the complete lack of symptoms and no early diagnostic markers, which is why more research is being directed towards the pathogenesis of the disease and it has been nearly a decade since the oncogenic processes driven in the Fallopian Tube (FT) is traced as the source for HGSC [9]. One of the determining evidence was the presence of Serous Tubal Intraepithelial Carcinoma (STIC) in the Fallopian tubes of around 60% of HGSC patients [10] and tubal ligation seemed to reduce the incidence of HGSC in patients showing BRCA1/BRCA2 mutations [9]. 

Non-coding RNAs (ncRNA) are a huge repertoire of RNAs, making up for nearly 98% of the transcriptome [11] that are transcribed from the genome but do not encode for proteins. Though their discovery began as early as 1955 through the discovery of ribosomal RNA (rRNA), the discovery of newer ncRNAs continues today [12]. As the number of ncRNAs discovered is increasing every day, there has not been a proportional increase in understanding their physiological roles. However, it is important to note that modern NGS techniques have decidedly broadened our understanding of their biological significance. In many cases, minor changes in RNA sequences are associated with changes in the expression of ncRNAs. A cell line wide genome analysis has allocated a cell type-specific expression profile of ncRNAs, suggesting their tissue specificity driving expression [12]. The ncRNA signature is very characteristic and depends on disease type; this is incidentally a very useful feature as it can help in not only identifying aberration in that particular tissue, but also could be used to target the disease in a multipronged approach. This could be crucial in diseases like ovarian cancer.

Since ncRNAs are increasingly observed to have important roles in disease as biomarkers, any newer paradigm for either diagnosis or therapy could go a long way in better management of EOC. Furthermore, HGSC being one of the most lethal cancers has also attracted several studies exploring the role of ncRNAs, specifically long non-coding RNAs in this context. Long non-coding RNAs (lncRNAs) are ncRNAs composed of >200 nucleotides, without evident open reading frames, and have versatile functions due to their ability to bind to both proteins and nucleic acids [13,14,15,16]. Because of their significant contribution to diagnosis, prognosis, and treatment, it would be useful to identify the ones that have an aberrant expression in ovarian cancer. Some of the lncRNA that is reported to be involved in ovarian cancer, with a specific role in HGSC are ANRIL, BC200, CCAT2, GAS5, H19, HOTAIR, Linc-RoR, MALAT1, MEG3, NEAT1, OVAL, PVT1, TUG1, UCA1, and XIST [17,18,19,20,21,22]. The repertoire of lncRNAs that are reported to play an important function in bringing about OC, specifically HGSC, is increasing day by day. Here, we add MAGI2-AS3 to this growing list, highlighting its role as a tumor suppressor, along with its possible signaling network in HGSC.

## 2. Results

### 2.1. Identification of Significantly Dysregulated Non-coding RNAs that Are Physiologically Relevant in HGSC

**Figure 1:**
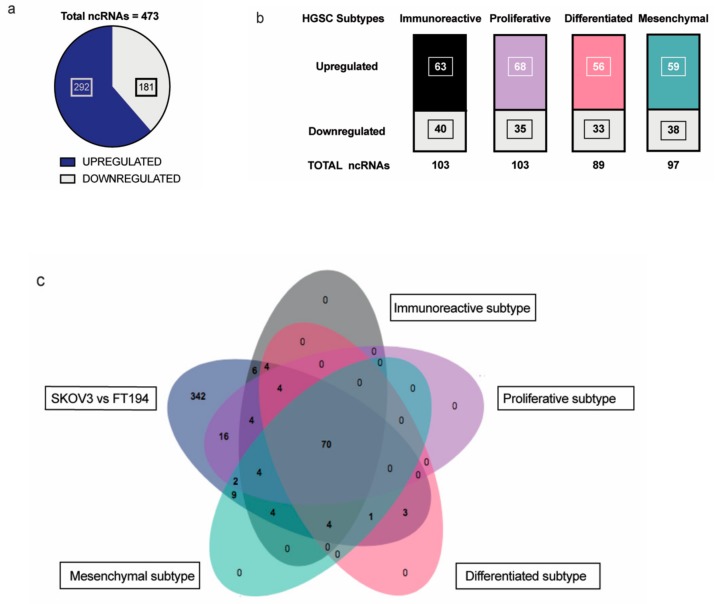
Analysis of RNA-seq results: (**a**) Distribution of significantly up and downregulated ncRNA in RNA-sequencing experiment between SKOV-3 and FT-194; (**b**) Up- and downregulated ncRNAs in each of the four HGSC subtypes—Immunoreactive, Proliferative, Differentiated, and Mesenchymal subtypes common with the RNA-seq experiment; (**c**) Venn diagram showing the distribution of ncRNAs detected from the differential expression analysis of the SKOV-3 vs FT-194 samples from RNA-seq experiment and the four HGSC subtypes versus Normal Fallopian tissue samples.

Non-coding RNAs are increasingly attributed to the disease specificity and being masters in genome regulation have crucial roles in the process of oncogenesis. This is of great significance especially in highly lethal ovarian cancers where there is much to be gleaned from pathogenesis of HGSC. Therefore, we wanted to identify the non-coding RNAs that could be possibly involved in tumorigenesis of EOC with respect to its tissue of origin, FT. Toward this, differential expression analysis was performed between SKOV-3 (Epithelial Ovarian Cancer cell line) and FT-194 (Fallopian Tube cell line) [23], using RNA sequencing data and several differentially regulated non-coding RNAs were identified by filtering for non-coding RNAs annotated in the Ensembl BioMart databank. A total of 473 non-coding RNAs (Supplementary Materials Table S1) were observed to be dysregulated between SKOV-3 and FT-194, out of which 181 were downregulated and 292 were upregulated in SKOV-3, as opposed to FT-194 represented in Figure 1a.

To identify non-coding RNAs that could play important physiological roles in EOC, a comparative transcriptome analysis was performed between ovarian cancer and normal fallopian tube tissues obtained from around TCGA-OV and GTEx samples, respectively. The data from TCGA-OV were separated into four HGSC subtypes—Proliferative, Immunoreactive, Mesenchymal, and Differentiated, as previously reported in [24] and further analyzed. Differential expression analysis of the four subtypes versus the Fallopian tube tissue samples shared several non-coding RNAs with our previous SKOV-3; FT-194 RNA-seq results indicated in Figure 1b. On comparing the two analyses, a total of 70 significantly differentially regulated non-coding RNAs were found to be common, as denoted in the Venn diagram from Figure 1c. These 70 commonly differentially regulated ncRNA are listed in Supplementary Materials Table S2.

**Figure 2:**
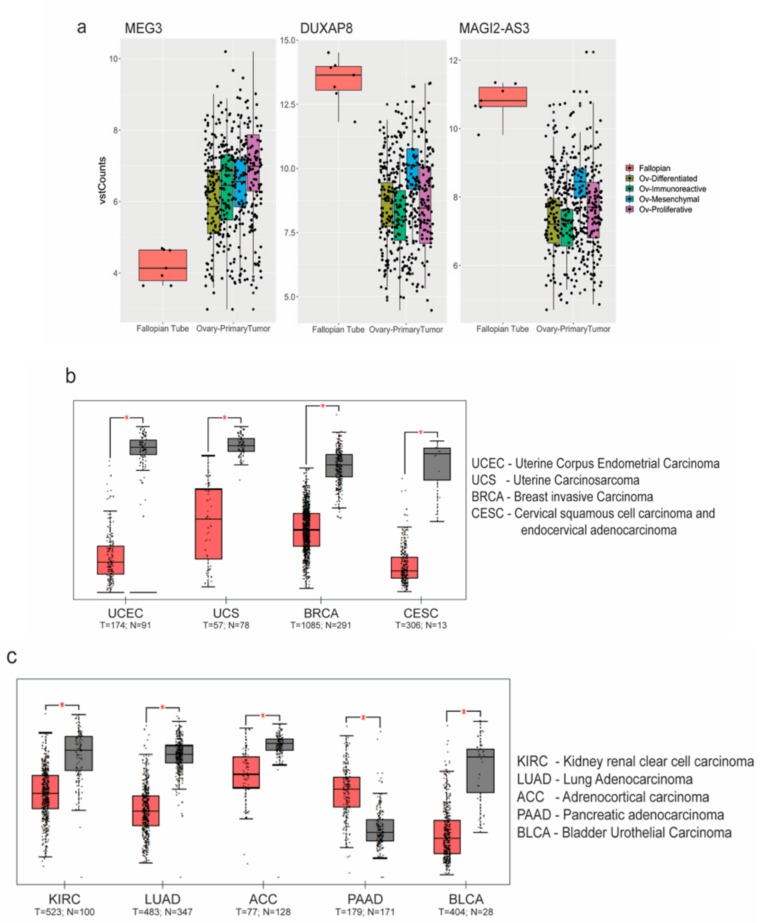
Analysis of non coding RNA (ncRNA) expression: (**a**) MEG3, DUXAP8 and MAGI2-AS3 ncRNAs plotted as box plots between High Grade Serous Ovarian Carcinoma (HGSC) subtypes and Fallopian Tube (FT) tissues obtained from The Cancer Genome Atlas - Ovarian Cancer (TCGA-OV) and Genotype Tissue Expression (GTEx-FT); Expression of MAGI2-AS3 in (**b**) gynecological; and (**c**) other obtained from TCGA using Gene Expression Profiling Interactive Analysis (GEPIA) (between tumor (red) and normal (grey) tissue).

To further confirm if the ncRNAs commonly regulated in both analyses displayed a similar trend, we chose three of the common ncRNAs—MEG3, DUXAP8, and MAGI2-AS3, and found the trend was validated in HGSC patient samples (from TCGA) with respect to FT (GTEx), as shown in Figure 2a. Amongst the significantly downregulated ncRNAs, MAGI2-AS3, a long non-coding RNA in the antisense strand of MAGI2 gene, was one of the top downregulated ncRNA based on fold change.

### 2.2. Expression and Regulation of MAGI2-AS3 in Epithelial Ovarian Cancer Cell Lines

Since the role of MAGI2-AS3 has not been previously explored in ovarian cancer, its function in this context was investigated. To understand if MAGI2-AS3 is uniquely dysregulated in ovarian cancers, the web server Gene Expression Profiling Interactive Analysis (GEPIA) [25] was used for analyzing MAGI2-AS3 expression in different cancers between normal and tumor tissues, as shown in Figure 2b,c. MAGI2-AS3 is consistently downregulated in all other gynaecological cancers other than ovarian cancer (Figure 2b). It is also observed to be downregulated in other cancers such as the rather aggressive pancreatic cancer, lung cancer, and kidney and adrenal cancers, as seen in Figure 2c. 

**Figure 3:**
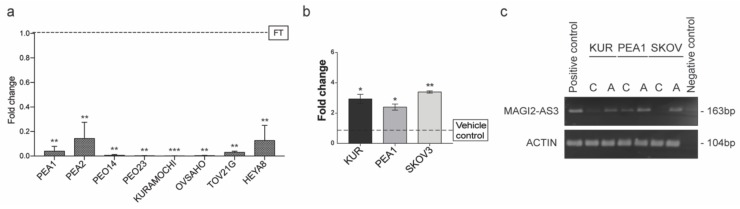
(**a**) Expression of MAGI2-AS3 in a panel of Epithelial Ovarian Cancer (EOC) cell lines with respect to FT (PEA1, PEA2, PEO14, PEO23, OVSAHO, KURAMOCHI, HeyA8 are HGSC cell lines; TOV21G is a Clear cell Ovarian carcinoma cell line and FT is Human Primary Fallopian Tube Epithelial culture); (**b**) Expression of MAGI2-AS3 72hrs after treatment of KURAMOCHI, PEA1 and SKOV3 with 10uM of 5-Aza-2′-deoxycytidine (5-AZA) and corresponding vehicle control is observed to increase; (**c**) Agarose gel electrophoresis image of endpoint PCR of MAGI2-AS3 and Actin used as housekeeping 72hrs after the treatment of KURAMOCHI, PEA1 and SKOV3 with 10uM of 5-AZA and corresponding vehicle control. For (**a**) and (**b**) the values are means ± SD of two independent experiments of real time PCR in duplicates normalized with respect to expression of house-keeping gene Abelson (ABL). *p*-value was calculated between control and test groups using t-test with cutoff as *p* ≤ 0.1.

The expression of MAGI2-AS3 was examined in a panel of EOC cell lines and primary human Fallopian tube cells using qPCR, as shown in Figure 3a. Of these, PEA1, PEA2, PEO14, PEO23, OVSAHO, KURAMOCHI, and HeyA8 are HGSC cell lines; TOV21G is a Clear cell Ovarian carcinoma (EOC) cell line and FT is Human Primary Fallopian Tube Epithelial cells. The expression of MAGI2-AS3 was negligible in all ovarian cancer cell lines with respect to the primary fallopian tube cells showing a consistent downregulation in EOC, suggesting its important role in this cancer. 

Often in cancers, the downregulation of a gene behaving as a tumor suppressor is regulated by epigenetic modifications [26]. An in-silico analysis on MAGI2-AS3 promoter region was performed using UCSC Browser, and two CpG islands were identified. Additionally, analysis using online software—DiseaseMeth 2.0 [27] revealed that MAGI2-AS3 promoter was hypermethylated in several cancers such as colon adenocarcinoma, head and neck carcinoma, uterine endometrial carcinoma, and rectal and anal adenocarcinoma. 

Based on these observations, it was hypothesized that MAGI2-AS3 could be downregulated epigenetically in EOC. Toward this, three EOC cell lines, PEA1, KURAMOCHI, and SKOV3 were treated with a demethylation inhibitor 5-Aza-2′-deoxycytidine (5-AZA) and the respective vehicle control for 72 h and the expression of MAGI2-AS3 after the treatment was checked using qPCR. As seen in Figure 3b, an increase in the expression of MAGI2-AS23 was observed after the inhibition of methylation, suggesting that the downregulation of MAGI2-AS3 is due to promoter hypermethylation. Figure 3c shows the agarose gel electrophoretic image of the 5-AZA treatment on EOC cell lines obtained after end point PCR of MAGI2-AS3, demonstrating an increased expression after treatment.

### 2.3. Expression of MAGI2-AS3 in EOC Cell Lines Decreases Their Adhesion to Extra Cellular Matrix, Migration, and Viability

**Figure 4:**
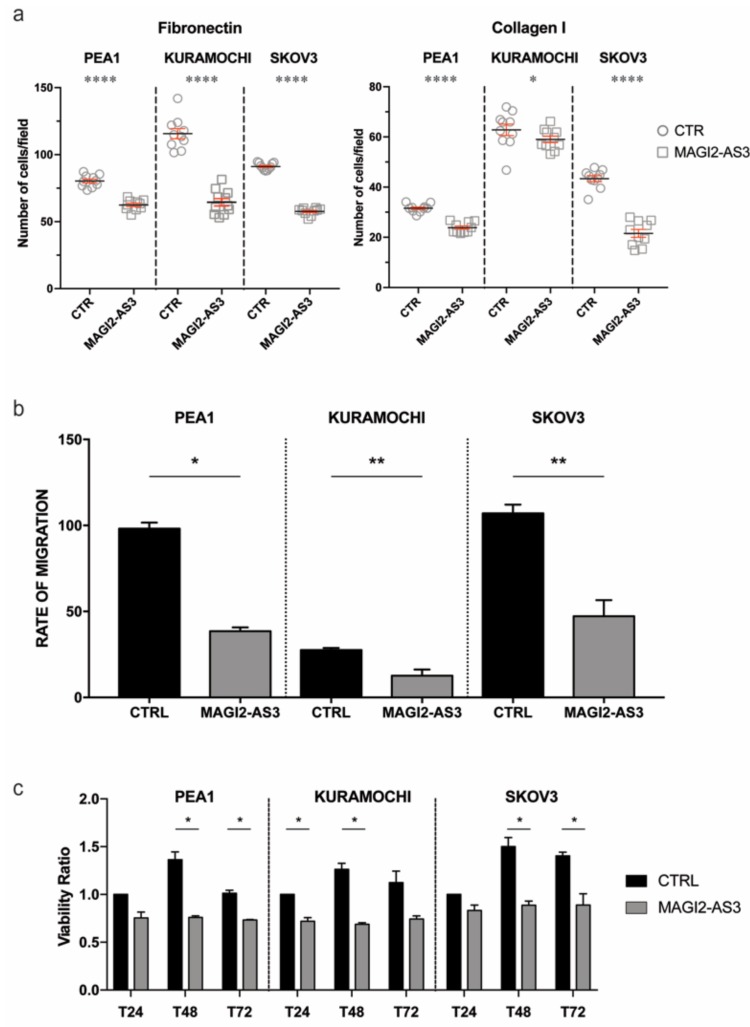
Effect of MAGI2-AS3 overexpression in EOC cell lines – PEA1, KURAMOCHI and SKOV3 after transfection with MAGI2-AS3 and control vector: on (**a**) adhesion to Fibronectin and Collagen I coated ECM substrates plotted as number of attached cells/ field (**b**) migration represented by cumulative rate of migration calculated across different time points (**c**) viability ratio between absorbance of control vector and that obtained at 24 h, 48 h and 72 h. The values are means ± SD of three independent experiments normalized with respect to the cells transfected with the control vector. *p*-value was calculated between control and MAGI2-AS3 transfected samples using *t*-test with cutoff as *p* ≤ 0.1.

To understand if the role of MAGI2-AS3 is consistent with that of a tumor suppressor, its physiological role in EOC cell lines was analyzed. Three EOC cell-lines were transfected with MAGI2-AS3 and its respective control vector (Supplementary Materials Figure S1) and the ability of the transfected cells to adhere to extra cellular matrix (ECM) mimicked by fibronectin or collagen coated coverslips was assessed. Figure 4a shows that MAGI2-AS3 expression significantly decreases the adhesion of EOC cell lines to both Collagen and Fibronectin ECM. 

The effect of MAGI2-AS3 expression on the migratory abilities of EOC cell lines was evaluated through wound healing assay after transfection with MAGI2-AS3 and control vector. Figure 4b demonstrates the reduction in the migratory abilities in the three EOC cell lines—PEA1, KURAMOCHI and SKOV3 attributed by the expression of MAGI2-AS3 with respect to the control. As observed in the figure, the rate of migration decreases in all the three OC cell lines upon MAGI2-AS3 expression.

Since MAGI2-AS3 was previously reported to be involved in the apoptotic pathway in breast cancer with Fas/FasL [28], the effect on the vitality of EOC by this lncRNA was checked by MTT assay. The viability of the EOC cell lines was evaluated after transfection with control and MAGI2-AS3 vectors at different time points, revealing (Figure 4c) that the expression of MAGI2-AS3 is capable of decreasing the viability of EOC cell lines.

Taken together, all of these experiments suggest that MAGI2-AS3 exerts a tumor suppressive role in EOC cell lines. 

### 2.4. MAGI2-AS3 Acts as A Competing Endogenous LncRNA in HGSC and Controls the Expression of miRNAs and Target mRNAs

**Figure 5:**
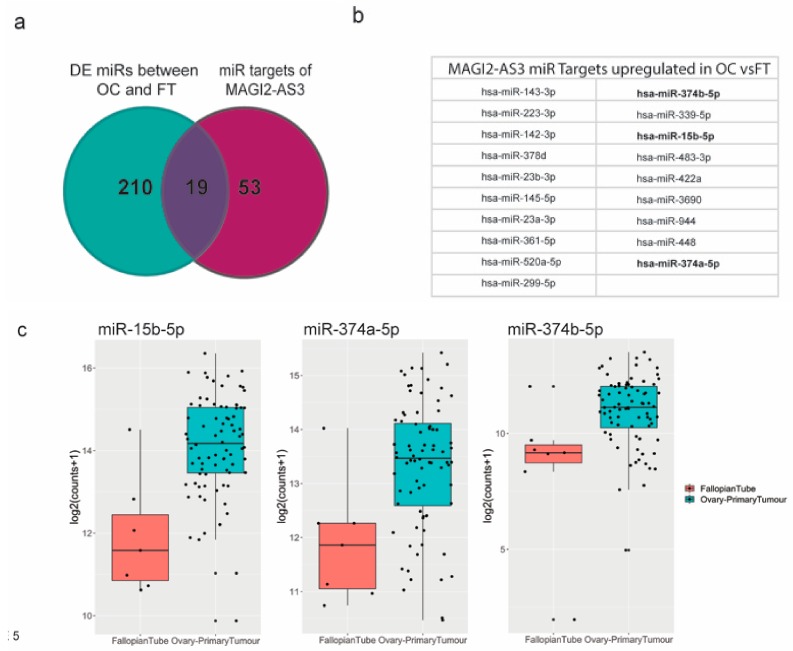
(**a**) miRNA targets of MAGI2-AS3 in Ovarian Cancer (OC) indicated through Venn diagram between miRNAs differentially expressed between OC vs. FT and all MAGI2-AS3 miRNA targets (**b**) List of miRNA targets of MAGI2-AS3 in OC that are common miRNAs between both the lists, of which 3 miRNAs chosen for further experimental validation are highlighted in bold (**c**) Expression of these 3 miRNAs- miR-15b-5p, miR-374a-5p and miR-374b-5p showing upregulation represented as box plots between FT (red) and OC (blue).

LncRNAs are commonly reported to have competing endogenous RNA function by sponging miRNAs in cytoplasm [16]. To identify MAGI2-AS3 miRNA targets in EOC, using Starbasev3.0 [29] and literature survey [30,31,32,33], a list of demonstrated and predicted miRNA targets was obtained. This was then matched with a list of all the differentially expressed miRNAs between EOC and FT [34,35]. Figure 5a shows a comparison of these two lists represented as a Venn diagram, and the common miRNAs obtained from both these lists are represented in Figure 5b.

Three miRNAs—miR-15b-5p, miR-374a-5p, and miR-374b-5p [30,31,32,33], highlighted in bold, were previously reported to be sponged by MAGI2-AS3 in other cancers, as shown in Supplementary Materials Table S3.

To understand if the mechanism of action of MAGI2-AS3 with respect to these miRNAs is similar in ovarian cancer (OC), the differential expression of these three putative miRNAs (Figure 5c) between EOC and FT data used in this study [36] was analyzed, and as expected, the expression of miR-15b-5p, miR-374a-5p, and miR-374b-5p was observed to be upregulated in EOC from FT, which is in accordance with the fact that they are MAGI2-AS3 ceRNA targets. 

**Figure 6:**
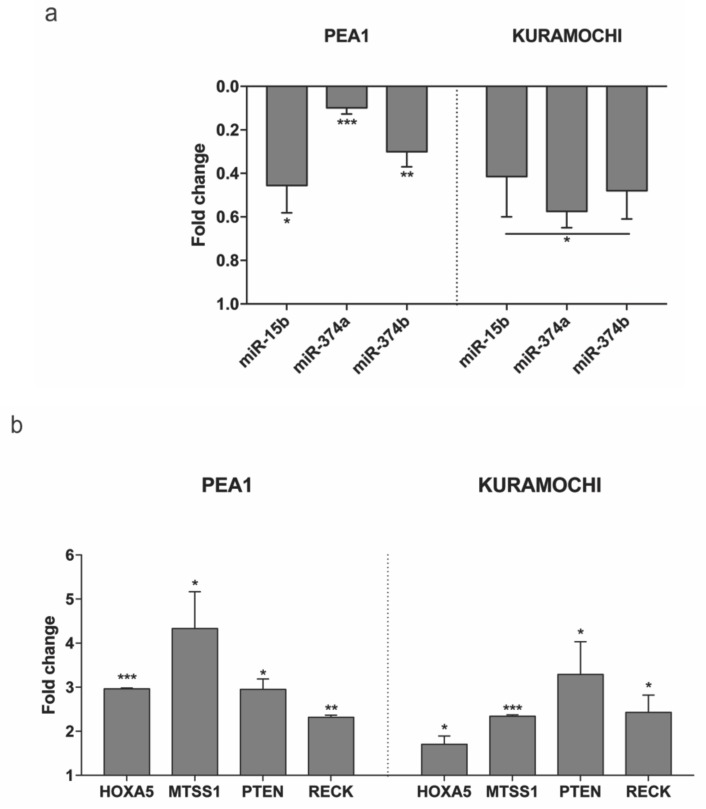
Expression of (**a**) the three miRNAs - miR-15b-5p, miR-374a-5p and miR-374b-5p and (**b**) the four mRNAs – HOXA5, MTSS1, PTEN and RECK evaluated after 24 h of transfection with MAGI2-AS3 vector with respect to control in HGSC cell lines – PEA1 and KURAMOCHI. The values are cumulative means ± SD of two independent experiments of real time PCR performed in duplicates normalized using house-keeping gene Abelson (ABL) with respect to the cells transfected with the empty vector. *p*-value was calculated between control and MAGI2-AS3 transfected samples using *t*-test with cutoff as *p* ≤ 0.1.

Subsequently, the expression of these miRNAs was checked in two HGSC cell lines—PEA1 and KURAMOCHI on transfection with MAGI2-AS3 and control vector. Figure 6a shows the decrease in expression of all three miRNAs upon MAGI2-AS3 introduction, suggesting that miR-15b-5p, miR-374a-5p and miR-374b-5p could be MAGI2-AS3 targets also in EOC, as observed in other cancers.

### 2.5. Expression of the Tumor Suppressor mRNAs Abrogated by miRNA in HGSC Can Be Restored by MAGI2-AS3 Introduction

miRNAs check the expression of specific proteins by complementary binding to their mRNA transcripts and cause their degradation [17]. Therefore, to further expand the ceRNA network of MAGI2-AS3, four mRNAs—HOXA5, MTSS1, PTEN, and RECK, reported to be associated with at least one of these three miRNAs—miR-15b-5p, miR-374a-5p, and miR-374b-5p (shown in Supplementary Materials Table S4) [37,38,39,40,41] and having oncosuppressive functions in OC [42,43,44,45] were selected. To determine their involvement in MAGI2-AS3 ceRNA network, the co-expression pattern of MAGI2-AS3 with each of the four mRNAs (HOXA5, MTSS1, PTEN, and RECK) in both OC (TCGA-OV) and FT (GTEx) was examined, as shown in Supplementary Materials Figure S2. The results show a highly significant and positive correlation between MAGI2-AS3 and the four mRNAs. Additionally, the expression of each of these mRNAs was downregulated in HGSC with respect to FT (Supplementary Materials Figure S3). Taken together, these bioinformatic analyses suggest that in the context of EOC, MAGI2-AS3 could plausibly regulate the expression of each of the four mRNAs (HOXA5, MTSS1, PTEN, and RECK).

To further experimentally confirm this interaction, the expression of each of the mRNAs upon introduction of MAGI2-AS3 was examined in HGSC cell lines, as shown in Figure 6b. The results show that the expression of HOXA5, MTSS1, PTEN, and RECK increases upon MAGI2-AS3 introduction, confirming that there is indeed an effect of MAGI2-AS3 on these mRNAs’ expression, which is possibly rescued by sequestration of the miRNA by the lncRNA. These results suggest that MAGI2-AS3, using its ceRNA activity, controls the expression of HOXA5, MTSS1, PTEN, and RECK in EOC. 

### 2.6. Identification of Enriched GO Bioprocesses and Construction of MAGI2-AS3 ceRNA Network in HGSC

The results previously mentioned suggest that MAGI2-AS3 elicits a physiologically tumor suppressive role in EOC, and also, using its role as a miRNA sponge, in turn regulates other mRNAs in HGSC. Since signaling pathways are actually complex networks, it is possible that MAGI2-AS3 regulates a variety of other biological processes apart from the ones shown above. 

**Figure 7:**
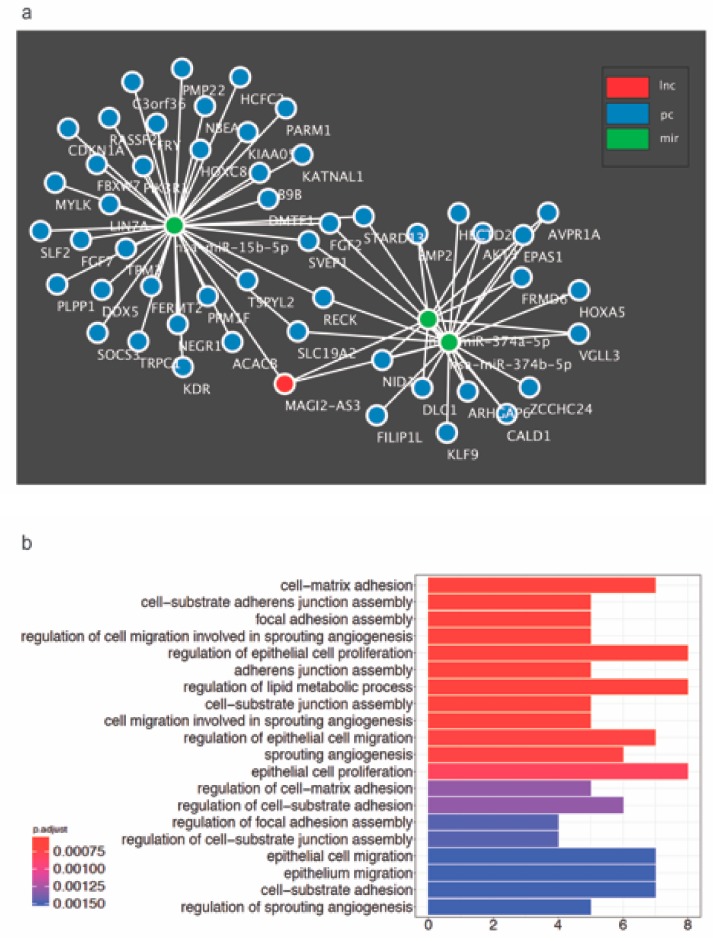
(**a**) Proposed competing endogenous RNA (ceRNA) network constructed bioinformatically for MAGI2-AS3 with its 3 target miRNAs – miR-15b-5p, miR-374a-5p and miR-374b-5p and their putative mRNA targets in HGSC (**b**) All Gene Ontology Biological process terms enriched by the MAGI2-AS3 ceRNA activity in HGSC obtained using GDCRNATools R-package.

To unravel newer mRNAs regulated by MAGI2-AS3, novel putative mRNA targets possibly regulated by MAGI2-AS3 were identified through the construction of a ceRNA network with MAGI2-AS3 sponges, miR-15b-5p, miR-374a-5p, and miR-374b-5p built using GDCRNATools is shown in Figure 7a. The putative mRNAs associated with each of the three miRNAs are indicated in the Supplementary Materials Figures S4–S6, while all the putative mRNAs used for the constructed of MAGI2-AS3 ceRNA network are listed in Supplementary Materials Table S5. This approach has hinted towards a larger signaling control directed by MAGI2-AS3 ceRNA network in HGSC, which has to be further probed. 

To further understand the role of MAGI2-AS3 in HGSC, the signaling pathways and processes enriched by MAGI2-AS3 in HGSC were determined bioinformatically. Towards this, all significantly downregulated putative and known mRNAs in HGSC versus FT targeted by the three miRNAs - miR-15b-5p, miR-374a-5p and miR-374b-5p sponged by MAGI2-AS3 were enriched for GO biological process terms as depicted in Figure 7b.

## 3. Discussion

Epithelial Ovarian Cancer (EOC), mainly attributed by High-Grade Serous Carcinoma (HGSC), is one of the most common gynecological malignancies and the fifth leading cause of cancer-related death in women [46]. It is typically diagnosed in advanced stages due to earlier symptoms and the mortality is due to its highly metastatic cancer characterized by widespread peritoneal dissemination and ascites accumulation [47]. Conventional chemotherapy, although appearing to work in the beginning, eventually fails, giving way to a relapse of the chemoresistant HGSC that causes the mortality of the patient.

Genomic analysis, previously performed by our group between EOC cell line SKOV3 and FT cell lines FT194, revealed another new important player in EOC: the non-coding RNA (ncRNA). ENCODE project observed that only 2% of the entire genome in protein-coding [11], while the remaining untranslated region is principally composed of non-coding RNAs, and more specifically long non-coding RNAs (lncRNAs) that attribute to disease-specificity. This aspect of lncRNA could have major value in better management of the aggressive HGSC. Toward this, a comprehensive analysis between RNA-sequencing previously performed, along with analysis of HGSC and FT transcriptomes from public databases, was done identifying the lncRNA MAGI2-AS3 to be significantly downregulated.

The first report showing experimental evidence of MAGI2-AS3 playing a tumor-suppressive role in breast cancer by regulating the Fas/FasL was published in 2018 [28]. Soon thereafter, other reports [31,33,48] demonstrated the tumor-suppressive function of MAGI2-AS3 in bladder cancer hepatocellular cancer and gliomas. Therefore, one of the first aims of this study was to understand if the onco-suppressive role of MAGI2-AS3 extends to EOC, specifically HGSC originating from FT. It is indeed interesting to note that MAGI2-AS3, by both bioinformatics and experimental methods, is observed to be consistently downregulated in EOC. It is also regulated in EOC cell lines through promoter hypermethylation like many other tumor suppressors, and further to this, its tumor-suppressive role using physiological assays like migration, invasion, and viability, is also corroborated in this study.

Recently, several reports have been published [30,31,32,33] showing lncRNA MAGI2-AS3 as a microRNA (miRNA) sponge to carry out its tumor-suppressive role in breast cancer, bladder cancer, hepatocellular cancer, and non-small cell lung cancer. Investigating along these lines, MAGI2-AS3 has been experimentally demonstrated to regulate the expression of three miRNAs—miR-15b-5p, miR-374a-5p, and miR-374b-5p that were previously associated with this lncRNA in other cancers [31,32,33]. It is indeed interesting to note that these three miRNAs that are upregulated in EOC [36] have been reported to have oncogenic roles in several other cancers [38,40,41,49,50,51,52,53,54,55,56,57,58]. It should be mentioned that some miRNAs, for example miR-15b-5p, have been observed to have conflicting roles as both oncogenes and tumor suppressors [49,50,59]. The miRNAs miR-374a-5p and miR-374b-5p have previously been reported to play a role in chemotherapy in pancreatic and ovarian cancer, respectively [53,60]. Also, MAGI2-AS3 was reported to have a prognostic value in glioma and shown to be involved in pathways regulating chemoresistance [61]. Therefore, taken together it is plausible that MAGI2-AS3 has important therapeutic relevance in HGSC and should be further pursued. If this is the case, epigenetic modifiers such as AZA already in clinical trials for ovarian cancer therapy [62] could effectively modulate the expression of MAGI2-AS3 to bring about tumor suppression.

This study highlights the numerous possibilities offered by the non-coding part of the genome in the development of HGSC and gives a glimpse of the pathway and the ceRNA network by which MAGI2-AS3 positively restricts the activity of HGSC cells. The mRNAs HOXA5, MTSS1, PTEN, and RECK are reported to have important tumor-suppressive roles in OC [42,45,63,64], though their role in HGSC is not specifically shown. This study has not only experimentally validated the regulation of the expression of tumor-suppressive mRNAs such as HOXA5, MTSS1, PTEN, and RECK by MAGI2-AS3 in HGSC but also has suggested a network of how various pathways converge to aid in tumor suppression. Each of these mRNA has been reported to have important functions in ovarian cancer with PTEN being the most noteworthy. PTEN loss, an important cancer driver, is among the earliest steps for HGSC development and has been correlated with two of MAGI2-AS3—miRNA targets used in this study; namely, miR-374a-5p and miR-374b-5p. In fact, two reports have shown the regulation of PTEN by MAGI2-AS3 ceRNA activity through a miR-23a-3p in non-small cell lung cancer [30] and miR-374a- 5p in breast cancer [32]. MTSS1, another mRNA regulated by this lncRNA has been well documented as a tumor suppressor controlling migration and invasion in OC with involvement in the EGF pathway [45]. To summarize, MAGI2-AS3 with its miRNA sponging action can bring about a cumulative onco-suppressive effect by its downstream target regulation. Therefore, it could be illuminating to further unravel other targets of MAGI2-AS3 from its extensive ceRNA network with a specific tumor-suppressive role in EOC.

Future studies should focus on unraveling specific non-coding RNA signature that could act as a prognostic and diagnostic tool for HGSC. In fact, MAGI2-AS3 should be further investigated for their therapeutic approach in combination with chemotherapy or after chemo-resistance as a last line of defense.

## 4. Materials and Methods

### 4.1. Differential Analysis of Coding and Long Non-coding RNAs

The RNA samples extracted from SKOV-3 (Epithelial Ovarian Cancer cell lines) and FT-194 (Fallopian Tube epithelial cell line) were sequenced by the Illumina HiSeq 1500 platform using a resolution at 100 base-pairs with paired-end reads. Analysis was performed using the RAP (RNA-Seq Analysis Pipeline) available on https://bioinformatics.cineca.it/97. The sequences quality check, the mapping, the transcriptomes assembling, and the differential expression analyses were performed using the default parameters on RAP. An alpha level of 0.05 was used for all the statistical tests. Gene expression data of significantly differentially regulated non-coding RNA was reported in Supplementary Materials Table S1.

RNA sequencing gene counts data of 430 samples of Ovarian Serous Cystadenocarcinoma from the TCGA-OV project and 7 healthy Fallopian tissue samples from GTEX were downloaded from the recount2 website (https://jhubiostatistics.shinyapps.io/recount/) [65]. The gene counts were scaled using the recount R package [34]. Ovarian cancer samples were further divided based on the four molecular subtypes—Mesenchymal, Immunoreactive, Proliferative, and Differentiated as defined in this report [35]. Pairwise differential expression analyses of the ovarian cancer subtypes versus the healthy Fallopian samples were performed using the Wald’s test available in the DeSeq2 R package [66]. Differentially expressed protein coding genes and non-coding RNAs with log2 fold-change ≥ |1| and an adjusted *p*-value ≤ 0.05 were extracted from these pairwise comparisons. Variance stabilizing transformation (vst) in DESeq2 was applied to the counts and the distribution of expression levels in the fallopian and ovarian cancer samples were visualized using box plots.

### 4.2. Expression of MAGI2-AS3 in Various Cancers

Differential expression analysis was performed between various cancers and their matched normal samples and was visualized using box plots with the online tool GEPIA (http://gepia.cancer-pku.cn) by setting log2 fold change ≥ |1| and *p*-value ≤ 0.01. The expression between normal samples (grey) from TCGA and GTEx and tumor samples (red) from TCGA were plotted in the log scale.

### 4.3. Venn Diagrams

All Venn diagrams were generated using the jVenn tool which is available online [67] (http://jvenn.toulouse.inra.fr/app/index.html).

### 4.4. Cell Lines and Culture Methods

The human ovarian cancer cell lines PEA1, PEA2, PEO14, and PEO23 were purchased from Sigma-Aldrich (St. Louis, Missouri, United States) and grown in RPMI-1640 medium supplemented with 10% fetal bovine serum, 2 mM glutamine, 2 mM sodium pyruvate, and 1% penicillin/streptomycin (Euroclone S.P.S., Pero, Italy). High-grade serous ovarian cancer cell lines KURAMOCHI and OVSAHO were obtained from the Japanese Collection of Research Bioresources Cell Bank (JCRB). The human ovarian cancer cell lines SKOV3, HeyA8 and TOV21G were provided by the CEINGE Cell Culture Facility (Naples, Italy). All these cell lines were maintained in RPMI-1640 medium supplemented with 10% fetal bovine serum and 1% penicillin/streptomycin (Euroclone). Immortalized Fallopian tube secretory epithelial cell line FT194 was provided by Dr. R. Drapkin (Boston, MA, USA). This cell line was grown in DMEM-F12 medium (Euroclone S.P.S., Pero, Italy) supplemented with 2% Ultroser G serum (PALL, Cergy-Saint-Christophe, France) and 1% penicillin/streptomycin. Primary human Fallopian Tube secretory epithelial cells were provided by Dr. U. Cavallaro (Milan, Italy) and was grown in appropriate medium [68].

### 4.5. Treatment with AZA

SKOV3, KURAMOCHI, and PEA1 cell lines were plated on the previous day and treated every 24 h with 10 μM of 5-Aza-2′-deoxycytidine,5-AZA (Sigma-Aldrich, St. Louis, MO, USA) and harvested after 72 h for RNA extraction.

### 4.6. Plasmid Preparation

The sequence of lncRNA MAGI2-AS3 transcript (NCBI Reference sequence - NR_038344.1) was amplified by PCR from FT194 cell line and constructed into pCDNA3.1 vector (Invitrogen, Carlsbad, CA, USA). The cloned vector was verified using sequencing provided by Eurofins Genomics service.

For the migration and adhesion assays, KURAMOCHI, SKOV3 and PEA1 cell lines were transfected using Lipofectamine 3000 (Invitrogen, Carlsbad, CA, USA) with control vector (pCDNA3.1) or MAGI2-AS3 using manufacturer’s protocol and harvested for RNA extraction 48 hours after transfection. 

For miRNA and mRNA analysis, PEA1 and KURAMOCHI cell lines were transfected using Lipofectamine 3000 (Invitrogen, Carlsbad, CA, USA) with control vector (pCDNA3.1) or MAGI2-AS3 using manufacturer’s protocol and harvested for RNA extraction 24 hours after transfection. 

### 4.7. RNA, cDNA and qRT-PCR

Total RNA was extracted using the RNeasy Mini kit (Qiagen, Hilden, Germany). The cDNA was synthesized using the iScript cDNA Synthesis kit (BIORAD, Hercules, CA). Real-time qPCR analysis was performed using the IQTM SYBR Green PCR Master Mix (BIORAD, Hercules, CA) in a CFX96 Real-Time PCR Detection System (BIORAD, Hercules, CA) for the following genes using gene-specific primers (Table 1):

For each gene, values are means ± SD of three independent experiments, normalized by the expression of housekeeping genes. To calculate the relative expression levels we used the 2-DDCT method [69].

### 4.8. Migration Assay

Migration assays were performed using Ibidi cell migration technology (Ibidi, Martinsried, Germany). SKOV3, KURAMOCHI, and PEA1 cell lines were transfected with MAGI2-AS3 and control vector (pCDNA3.1) as described before. After 24 hours, both control and lncRNA transfected cells were seeded in each chamber at a density of 3 × 10^5^ cells/reservoir in 70 μL of normal medium for 24 h. The medium was then replaced with fresh medium and the cells were treated with 10 μg/mL of Mitomicyn C (Sigma-Aldrich, St. Louis, MO, USA) for 1 h at 37°C. After the incubation, the chambers were removed and cells were further incubated in normal medium. Cells were photographed (1:1 magnification) from T_0_ to T_end_ (8 h for PEA1, 32 h for KURAMOCHI and 8 h for PEA1) at regular intervals (2 h each for PEA1 and SKOV3 and 8 h for KURAMOCHI), and the distance covered by cells within a defined area in the gap measured using NIH ImageJ (rsb.info.nih.gov/ij) software. The rate for each time point was calculated for MAGI2-AS3 and control transfected and the results are the average of 3 experiments plotted using GraphPad Prism software Version 7.0a (GraphPad Software, SD, USA).

### 4.9. Adhesion Assay

Coverslips were coated with Fibronectin (10 µg/mL; Calbiochem, San Diego, CA, USA) or Collagen I (10 μg/mL; Invitrogen, Carlsbad, CA, USA) in PBS 1X for 1 h at 37 °C. SKOV3, KURAMOCHI and PEA1 cell lines were transfected with control pCDNA3.1, MAGI2-AS3 and HAND2-AS1 vectors as described before. After 48 hours, 40 × 10^3^ of control and lncRNA transfected cells were plated on top of coated coverslips in triplicates for 2 hours at 37°C. After incubation, the coverslips were washed with PBS 1X, fixed in 4% paraformaldehyde for 10 min and stained with HOECHST. The experiment was repeated three times (n = 3) for each cell lines. Images were acquired using a Confocal microscope (LSM 700 ZEISS, 73447 Oberkochen, Germany). For each coverslip, 10 images were acquired and analyzed using ImageJ software. The results plotted are the average of 3 experiments with values normalized and transformed using formula Y = log(Y) using GraphPad Prism software Version 7.0a (GraphPad Software, San Diego, USA).

### 4.10. Viability Assay

To assess the viability, SKOV3, KURAMOCHI, and PEA1 cells were transfected with both control pCDNA3.1and MAGI2-AS3 vectors. 24 h after transfection 1 × 10^4^ cells per sample were plated in triplicate on 96-well plates under regular culture conditions. Cell viability was detected 24 h, 48 h, and 72 h later using the MTS reagent (Promega, Madison, WI, USA) and represented as T24, T48 and T72. The viability ratio of cells grown in the two different wells was calculated using OD_sample well_/OD_control well_ where control well is that with control vector at time T0.

### 4.11. Differential miRNA Analysis

Differentially expressed miRNAs between high grade ovarian carcinoma and normal fallopian tissue were obtained from the study performed by Chen, et al. [36]. Normalized log transformed expression data used in this study were downloaded from the Gene Expression Omnibus, GSE65819 [35] and used for the comparison of expression of hsa-miR-15b-5p, hsa-miR-374a-5p and hsa-miR-374b-5p in ovarian cancer and normal fallopian samples (Figure 5).

### 4.12. miRNA Extraction, RT and qPCR

miRNA was extracted using the miRNeasy Mini Kit (Qiagen, Hilden, Germany). The cDNA was synthesized using the miScript II RT kit (Qiagen, Hilden, Germany). Real-time qPCR analysis was performed using the miScript SYBR Green PCR kit (Qiagen, Hilden, Germany) in a CFX96 Real-Time PCR Detection System (BIORAD, Hercules, CA, USA) for the miRNAs—MiR-15b-5p, miR-374a-5p, miR-374b-5p using primers specifically designed with miScript Primer Assays (Qiagen, Hilden, Germany).

### 4.13. Correlation Plots

Gene expression correlation plots (Pearson correlation) for MAGI2-AS3 against RECK, PTEN, HOXA5, and MTSS1 genes, for TCGA-OV tumor and Fallopian tissue normal samples were obtained from GEPIA (Supplementary Materials Figure S2).

### 4.14. miRNA-mRNA Targets

mRNA targets of hsa-miR-15b-5p, hsa-miR-374a-5p and hsa-miR-374b-5p were obtained from two databases: 1) starBase with the following filters: > 5 pan cancer, high fidelity > 3 clip seq data, detected in > 2 programs and 2) mirTarbase [20,21,22,23,24,25,26,27,28,29,30,31,32,33,34,35,36,37,38,39,40,41,42,43,44,45,46,47,48,49,50,51,52,53,54,55,56,57,58,59,60,61,62,63,64,65,66,67,68,69,70]. The mRNA targets which were common with protein coding genes down-regulated in at least three ovarian cancer subtypes with respect to the normal fallopian tube samples were extracted. Gene Ontology (GO) Biological process and KEGG pathway enrichment using these targets were performed with the clusterProfiler package in R [71] (Supplementary Materials Figures S4–S6).

### 4.15. MAGI2-AS3 ceRNA Network Prediction

374 RNAseq and 489 miRNAseq TCGA-OV primary tumor samples were downloaded and pre-processed using the GDCRNATools R package [72]. 

For MAGI2-AS3 ceRNA network, the combined list of mRNA targets of hsa-miR-15b-5p, hsa-miR-374a-5p and hsa-miR-374b-5p obtained previously from starBase and miRTarBase [20,21,22,23,24,25,26,27,28,29,30,31,32,33,34,35,36,37,38,39,40,41,42,43,44,45,46,47,48,49,50,51,52,53,54,55,56,57,58,59,60,61,62,63,64,65,66,67,68,69,70] were used for ceRNA analysis with GDCRNATools (Supplementary Materials Table S11). 

lncRNA-miRNA-mRNA interactions with a hyperPValue < 0.05 and corPValue < 0.05 were used to build the ceRNA network for ovarian cancer. The network was then visualized using Cytoscape V3.7.1 [73] (Figure 7b). The protein coding genes in the ceRNA network were enriched for GO Biological process and KEGG pathway terms (Figure 7a).

### 4.16. Figure for Graphical Abstract:

Biorender app (https://biorender.com) was used to generate the graphical abstract.

## 5. Conclusions

This study for the first time highlights the tumor-suppressive role of LncRNA MAGI2-AS3 in HGSC and demonstrates its involvement in the regulation of miR-15b-5p, miR-374a-5p, and miR-374b-5p, and of their downstream mRNA targets (HOXA5, MTSS1, PTEN, and RECK) in HGSC cell lines. Moreover, we propose a ceRNA network for MAGI2-AS3 in HGSC, which has to be further explored.

## Figures and Tables

**Table 1:** Gene-specific Primers.

ABL fwd	5’- TGGAGATAACACTCTAAGCATAACTAAAGG -3′
ABL rev	5′- GATGTAGTTGCTTGGGACCCA-3′
MAGI2-AS3 fwd	5′- TCTTCAAGAGCCAGGGACAG -3′
MAGI2-AS3 rev	5′-TGCAGCTCAAACTCTCCAGA -3′
HOXA5 fwd	5′- CAACCCCAGATCTACCCCTG -3′
HOXA5 rev	5′- TTCAATCCTCCTTCTGCGGG -3′
MTSS1 fwd	5′- CCTCAGTTGGACAGTGCTCT -3′
MTSS1 rev	5′- GCAGTTTGTGAGGGTCCATG -3′
PTEN fwd	5′- AAGCTGGAAAGGGACGAACT -3′
PTEN rev	5′- TACACATAGCGCCTCTGACT -3′
RECK fwd	5′- CAGACTCTTCTCCTGGTCCA -3′
RECK rev	5′- TCAGGATTCTCTTGCAGGCA -3′

## Supplementary Materials

The following are available online at https://www.mdpi.com/2072-6694/11/12/2008/s1, Table S1: List of significantly regulated non-coding RNA between FT194 and SKOV3, Table S2: List of ncRNAs from RNA-seq common with HGSC(TCGA-OV) vs FT(GTEx), Figure S1: Expression of MAGI2-AS3 after transfection in EOC cell lines, Table S3: miRNAs associated with MAGI2-AS3 in other cancers, Table S4: mRNA targets of the three miRNAs chosen for experimental analysis, Figure S2: Correlation plots between MAGI2-AS3 and mRNAs, Figure S3: Expression of mRNAs between HGSC (TCGA-OV) and FT (GTEx), Figure S4: Analysis between miR-15b-5p and its putative mRNA targets, Figure S5: Analysis between miR-374a-5p and its putative mRNA targets, Figure S6: Analysis between miR-374b-5p and its putative mRNA targets, Table S5: Putative mRNAs associated with miR-15b-5p, miR-374a-5p and miR-374b-5p in HGSC used to construct ceRNA network
