# Supplementary material for: Long Non-Coding RNA MAGI2-AS3 is a New Player with a Tumor Suppressive Role in High Grade Serous Ovarian Carcinoma
Source: Cancers (Basel). 2019 Dec 12;11(12):2008. doi: 10.3390/cancers11122008 (PMC6966615; doi:10.3390/cancers11122008)
Supplement: Supplementary file 1 [file cancers-11-02008-s001.zip › Supplementary files revised/Supplementary Table S4.docx]

| miRNA | mRNA Targets |
| --- | --- |
| miR-15b-5p | MTSS1, RECK |
| miR-374a-5p | PTEN |
| miR-374b-5p | HOXA5, PTEN, RECK |
