# Supplementary figures and images for: Long Non-Coding RNA MAGI2-AS3 is a New Player with a Tumor Suppressive Role in High Grade Serous Ovarian Carcinoma
Source: Cancers (Basel). 2019 Dec 12;11(12):2008. doi: 10.3390/cancers11122008 (PMC6966615; doi:10.3390/cancers11122008)

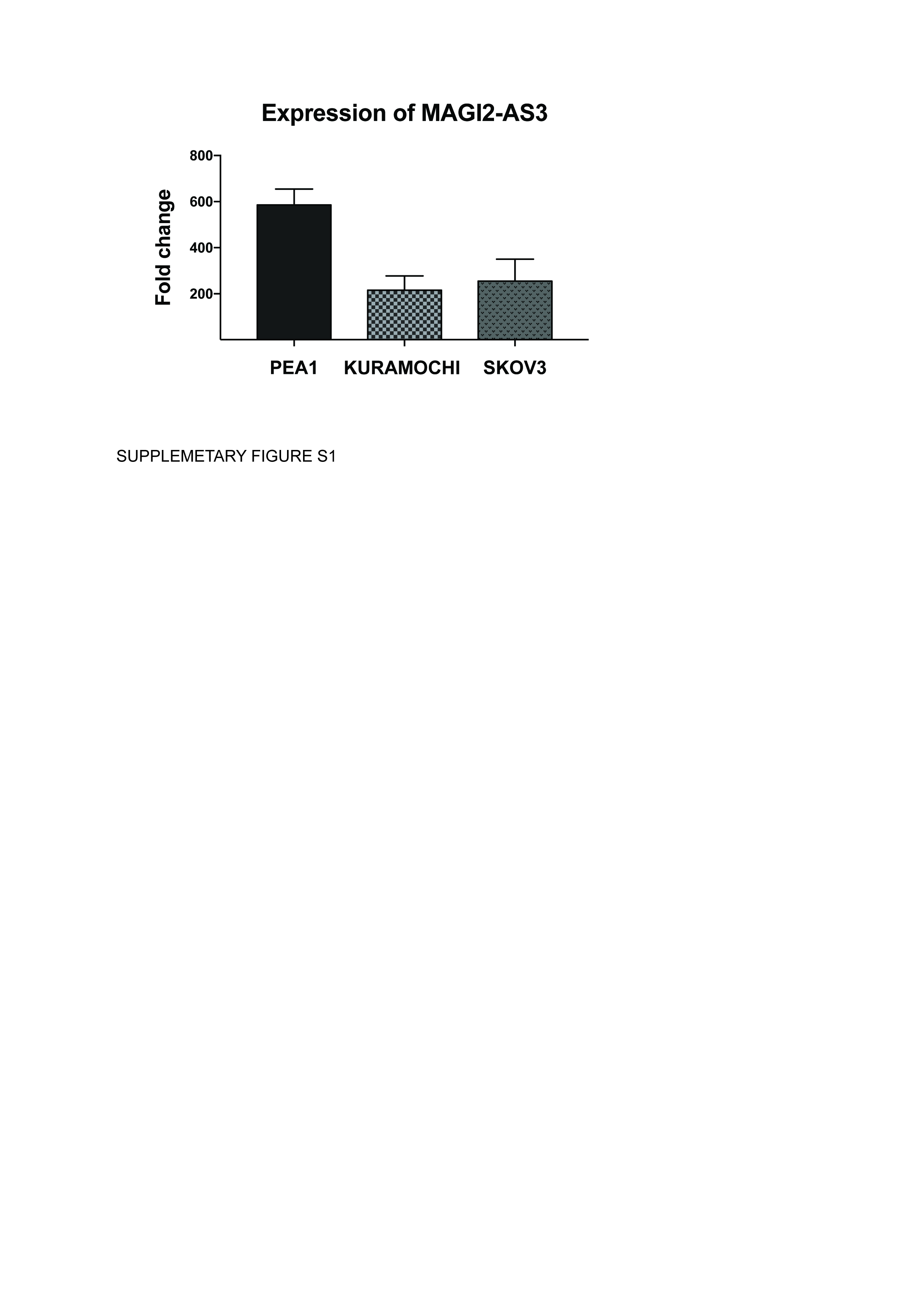

Supplement: Supplementary file 1 [file cancers-11-02008-s001.zip › Supplementary Figure S1.tif]

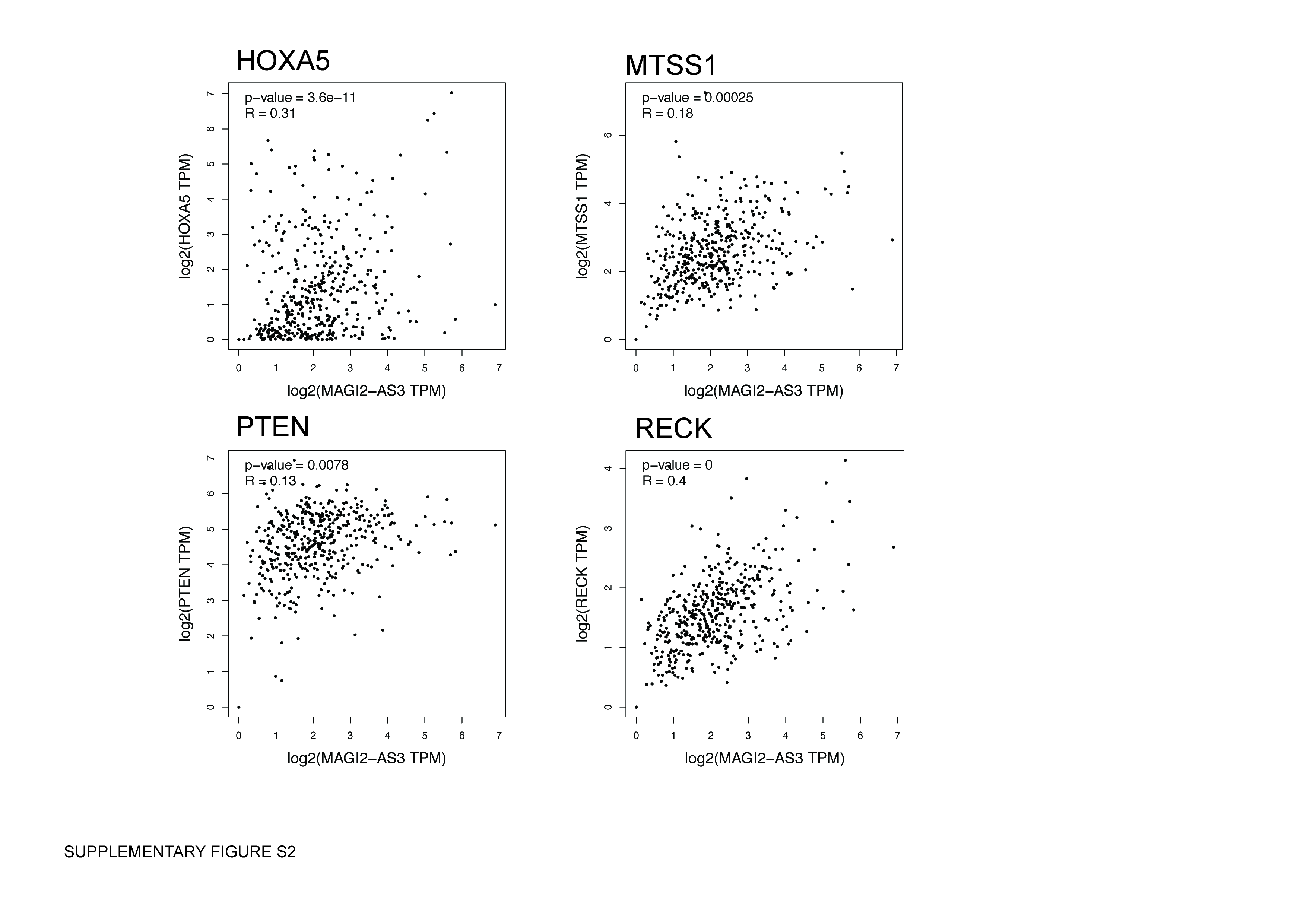

Supplement: Supplementary file 1 [file cancers-11-02008-s001.zip › Supplementary Figure S2.tif]

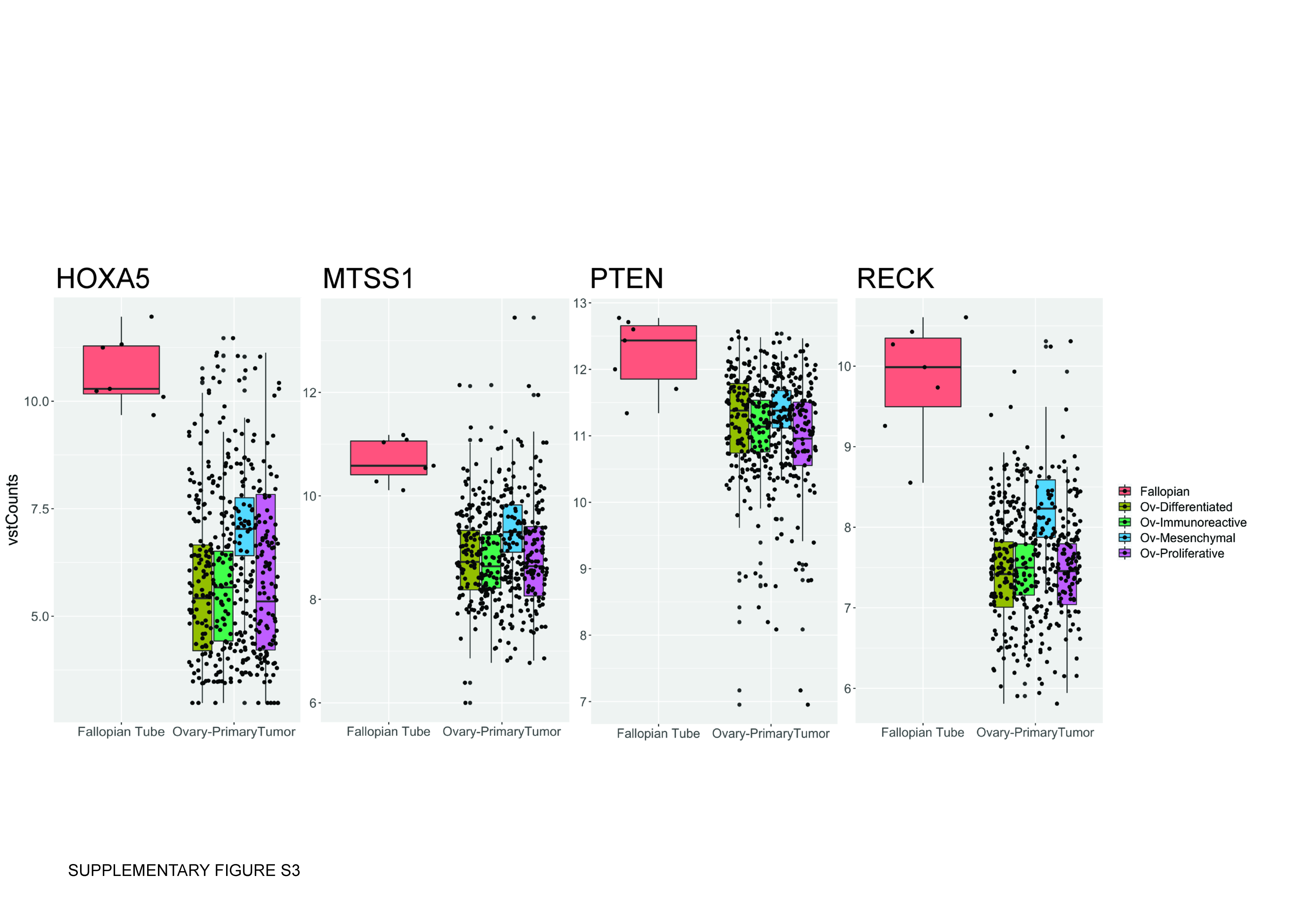

Supplement: Supplementary file 1 [file cancers-11-02008-s001.zip › Supplementary Figure S3.tif]

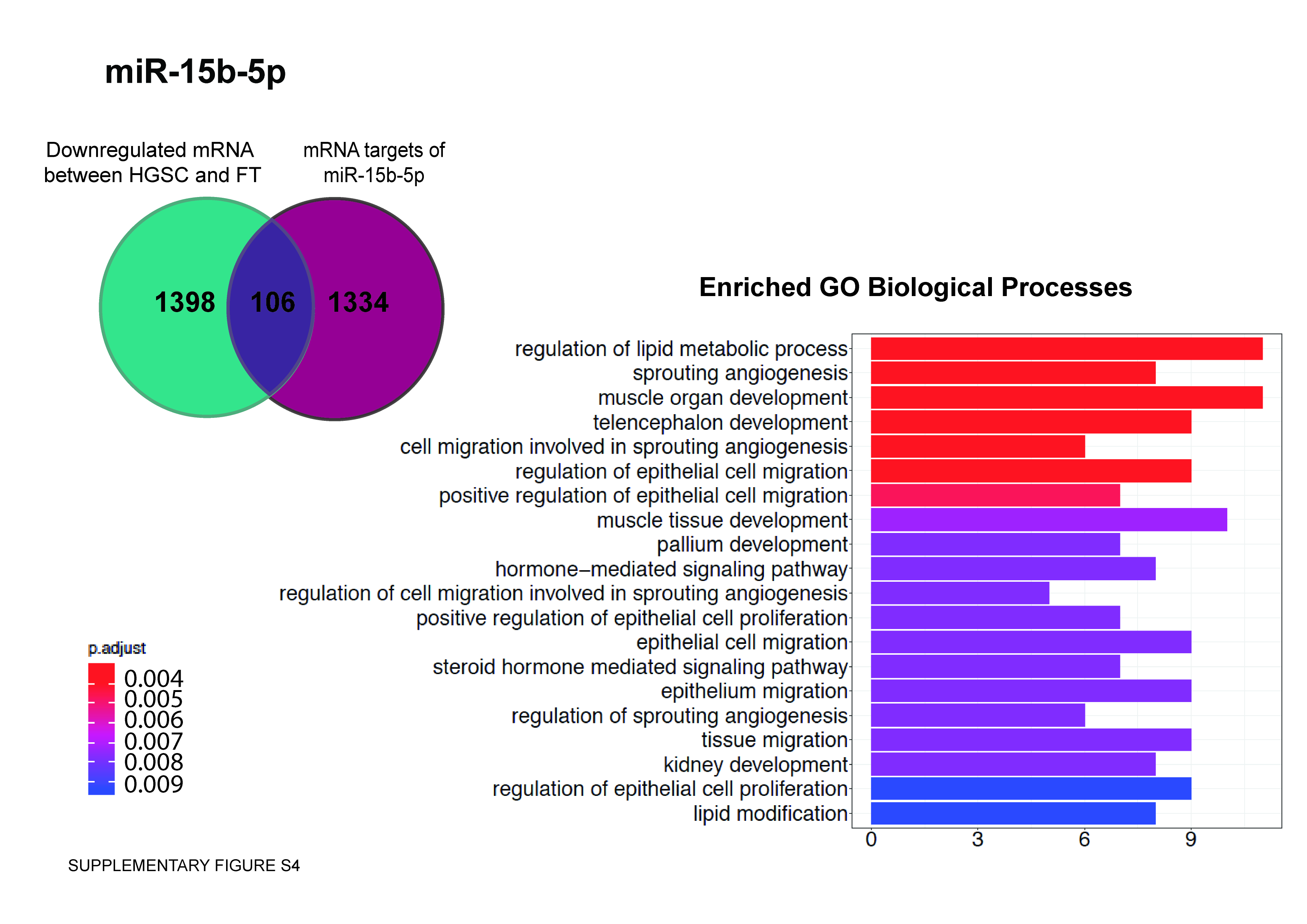

Supplement: Supplementary file 1 [file cancers-11-02008-s001.zip › Supplementary Figure S4.tif]

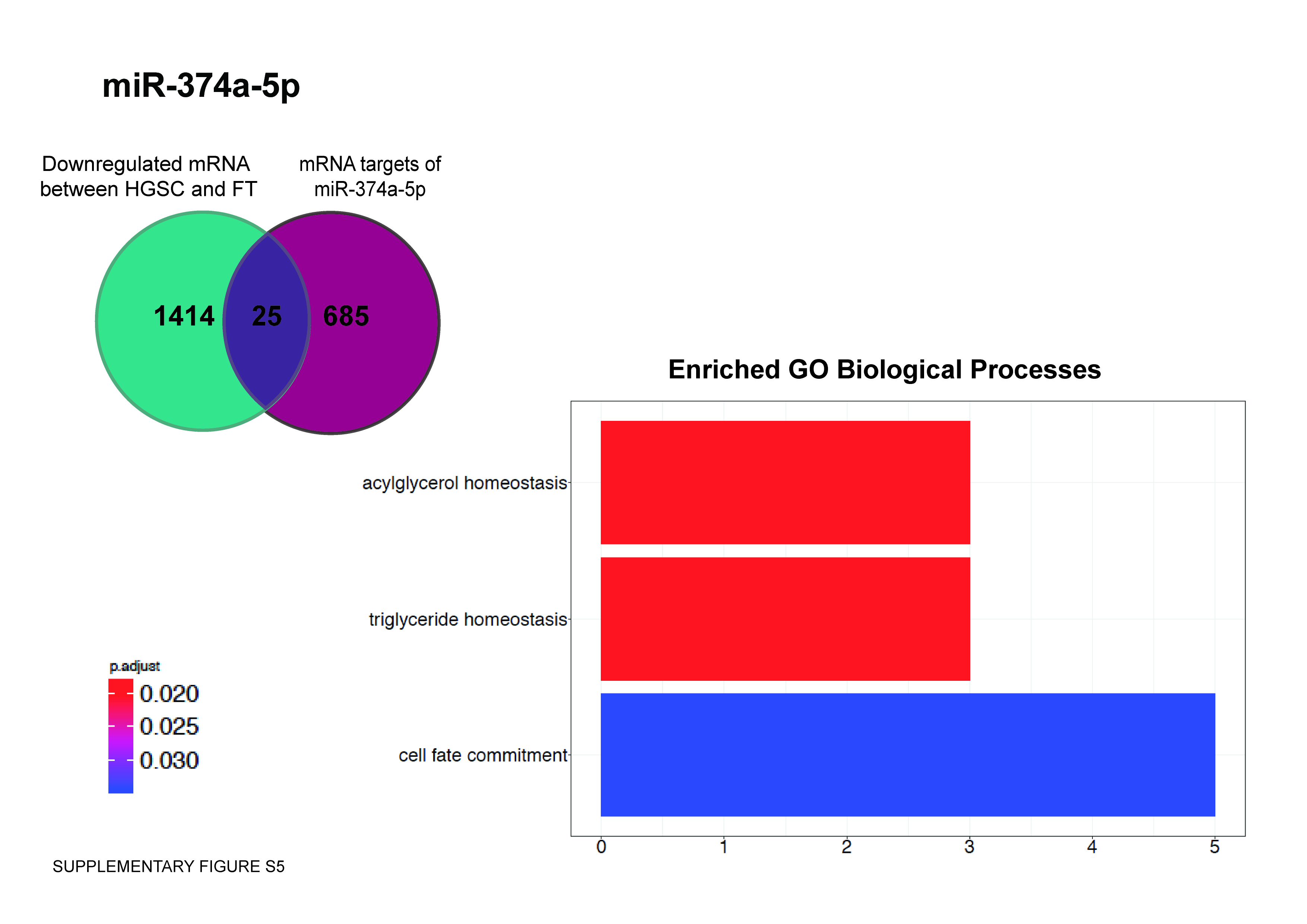

Supplement: Supplementary file 1 [file cancers-11-02008-s001.zip › Supplementary Figure S5.tif]

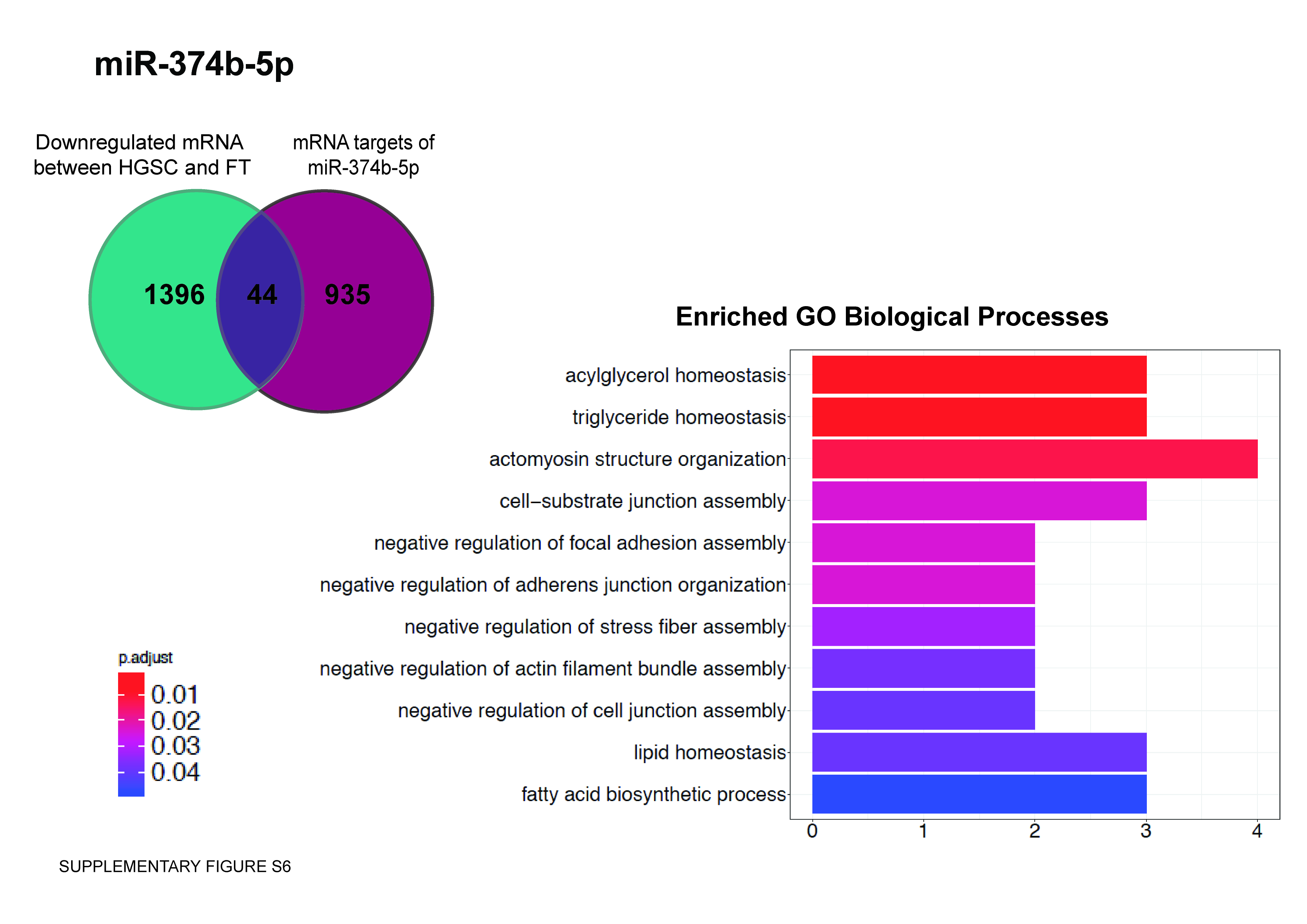

Supplement: Supplementary file 1 [file cancers-11-02008-s001.zip › Supplementary Figure S6.tif]
